# Inhibition of Notch 1 receptor influenced the differentiation of Lin-CD45RA-dendritic cell precursors within ovarian carcinoma microenvironment

**DOI:** 10.1186/s12865-016-0150-3

**Published:** 2016-06-04

**Authors:** Xue-qian Qian, Li-li Chen, Qi Cheng, Yang Tian, Xiao-feng Luo, Xiao-yun Wan

**Affiliations:** Women’s Hospital, School of Medicine, Zhejiang University, Xueshi Road 1#, Hangzhou, China; Department of Gynecologic Oncology, Women’s Hospital, School of Medicine, Zhejiang University, Hangzhou, China; The First Affiliated Hospital, School of Medicine, Zhejiang University, Qingchun Road 79#, Hangzhou, China; Key Laboratory of Combined Multi-organ Transplantation, The First Affiliated Hospital, School of Medicine, Zhejiang University, Hangzhou, China

**Keywords:** Ovarian carcinoma, Dendritic cell(DC), Myeloid dendritic cell (mDC), Plasmacytoid dendritic cell (pDC), Precursor, Notch1

## Abstract

**Background:**

Previous evidence suggested that the differentiation of Lin-CD45RA-DC precursors were prior to plasmcytoid dendritic cells (pDCs) than myeloid dendritic cells (mDCs) within ovarian cancer microenvironment. However, the mechanism is still unclear. Therefore, we investigated the function of Notch 1 signal pathway in the differentiation of Lin-CD45RA-DC precursors.

**Methods:**

The CD34+ hematopoietic stem cells were extracted from umbilical cord blood in term parturition, and Lin-CD45RA-DC precusors were separated and induced mature. Expression of Notch1 receptor and ligands in Lin-CD45RA-DC precusors was detected by Real-time PCR and was down-regulated by shRNA or γ-secretase inhibitor (GSI). Flow cytometry was used to analyze the subset of DCs with or without SKOV3 culture supernatants. IL-12 level was detected by ELISA.

**Results:**

Expression of Notch1 receptors and ligands were detected in Lin-CD45RA-DC precursor cells. The Notch1 mRNA in Lin-CD45RA-DC precursors can be down-regulated by shRNA-Notch1 lentivirus transfection and GSI. ShRNA mediated Notch 1 knock-down significantly differentiated less plasmcytoid dendritic cells (pDCs), but generated more myeloid dendritic cells (mDCs), and this would not be influenced by the supernatant of the ovarian carcinoma cell line. GSI had the same effect in the differentiation of pDC. The secretion of IL-12 significantly increased after Notch1 knock-down with or without SKOV3 culture supernatants.

**Conclusions:**

Notch1 is an important signaling pathway in the differentiation of Lin-CD45RA-DC precursor cells to plasmcytoid dendritic cells (pDCs). And this would not be affected by the supernatant of the ovarian carcinoma cell line.

## Background

Ovarian epithelial carcinoma is one of the most common gynecological malignancies encountered in the clinic. It has a biological behavior of metastasis initiated at an early stage and rapidly spread over the peritoneal cavity. Published literatures revealed that the immune deficiency in tumor microenvironment plays an important role in the pathogenesis of ovarian cancer, and also influences the disease progression, and overall survival rate [1–3]. One evidence to support the above statement is that the matured myloid DCs (mDCs, HLA-DR + CD11C + CD123−), which are considered as the most potent antigen-presentation cells, infiltrate rarely into the tumor site in the patients with ovarian carcinoma. Of note, mDCs play an important role in promoting immune response, while the plasmcytoid DCs (pDC, HLA-DR + CD11C − CD123+) are oppositely to promote immune escape [4–7].

It is known that mDC precursors would probably present as Lin-CD45RA- cells because no specific surface markers such as CD45RA were expressed and lineage negative. However, each DC subtypes have the potential ability to differentiate through either myeloid or lymphoid-lineages and the DC development is quite flexible rather than their lineage restriction [8, 9]. In our previous study, we cultured such precursors that generated from CD34+ human progenitor cells (HPC) with GM-CSF and TNF-α in the ovarian carcinoma microenvironment. Then, we observed that the Lin-CD45RA- DC precursors differentiated into more pDCs than mDCs, which indicated that it may favor tumor progression [10]. However, the mechanism is still unclear.

Notch proteins (Notch 1-4) constitute a group of highly conserved transmembrane receptors that regulate cell fate decision during the development of many mammalian cell lineages. A lot of studies have provided much data on the critically important role of the Notch pathway in the differentiation of myeloid cells. Recent studies also confirmed the expression of Notch protein in antigen presentation cells and matured DC [11]. More and more papers concluded that Notch pathway is associated with DC differentiation. But the conclusions were controversial [12]. Cheng et al. described that the differentiation of DC in Notch-1 anti-sense mice was significantly down-regulated that only half of the normal level of Notch-1were expressed in HPC [13]. Yet, Radtke et al. found that in Notch-1 conditional knockout mice, the thymic DCs, conventional DCs, and Langerhans cells were in normal level [14].

Based on our previous research works, the current study is aimed to address the expression of Notch1 in Lin-CD45RA-DC precursors, and then to illustrate the relationship between Notch1 and differentiation of the precursors in vitro.

## Methods

### Ovarian carcinoma cell line culture and collection of supernatants

An epithelial ovarian carcinoma cell line SKOV3 was purchased from American Type Culture Collection. SKOV3 was cultured in McCOY'5A (Gibco BRL, USA) with 15 % FBS and passaged when confluent with 0.25 % trypsin. When cells grew to about 80 % of bottle plat, the medium was changed by RPMI 1640 supplemented with 15 % FBS for 36 h. The supernatants were collected for future use.

### Isolation and enlargement of Lin-CD45RA-DC precursors

Normal human cord blood was collected immediately from the full term deliveries, according to the Institution Guideline. Informed consents were obtained from all volunteers. CD34+ HPCs were isolated from cord blood by magnetic cell separation (MACS, Miltenyi Biotec, Germany) with the use of anti-CD34 − mAb-conjugated immunomagnetic beads (Miltenyi Biotec, Germany) according to the manufacturer’s instructions. More than 95 % of the isolated cells expressed CD34 when analyzed by flow cytometry (data not shown). Enriched CD34+ HPC (with initial density of 5 × 10^5^/ml) were cultured in RPMI 1640 supplemented with 15 % FBS, penicillin (100 U/ml), and streptomycin (100 mg/ml). Cells were maintained at 37 °C in a 5 % CO2 humidified atmosphere for 7 days in the presence of recombined human cytokines (R&D system, USA) FLT-3L (50 ng/ml) and SCF (50 ng/ml), could only expand but not influence the differentiation of the precursors[15, 16], for enlargement. Half the culture medium and cytokines were renewed every 3 days. Expanded progenitor cells were then collected for sorting DC precursors. In brief, cells were incubated with (FITC)-conjugated Lineage cocktail 1 (Lin 1, BD Biosource, USA) for 10 min in 4 °C. After washed with buffer solution and incubated with anti-FITC microbeads (MiltenyiBiotec, Germany) for 20 min in 4 °C, cells were isolated by automatic MACS. Lin 1 contains monoclonal antibody (mAb) clones against CD3 (T cells), CD14 (monocytes/macrophages), CD16 (natural killer cells), CD19 (B cells), and CD20 (B cells), CD56 (natural killer cells). After isolation all these cells were removed. Then by using CD45RA microbeads (MiltenyiBiotec, Germany), Lin-CD45RA- cells were obtained. More than 95 % of these isolated cells were Lin-CD45RA- when analyzed by flow cytometry.

### Quantitative real-time PCR

RNA expression was measured by using a Quantitative Real-Time PCR (qRT-PCR ) assay with the SYBR Green I dye. The housekeeping gene, GAPDH, was used as an internal control. After cells were collected, total RNA was extracted from Lin- CD45RA- cells by using TRIzol reagent (TaKaRa, Dalian, China). cDNA was synthesized by a PrimeScript RT reagent kit (TaKaRa, Dalian, China). The mRNA-specific primers were designed with the Lasergene software (DNASTAR, Madison, WI) and span at least one intron with an average length > 800 bp. PCR experiments were conducted on an MX3000P real-time PCR thermal cycler using software version 4.01 (Stratagene, La Jolla, CA). PCR products were electrophorized on 2 % (w/v) agarose gels. All qRT-PCR measurements were conducted in triplicates. The relative mRNA expression was calculated using the △△Ct method. The sequences of the primers for real-time PCR are listed in Table 1.

**Table 1:** List of gene-specific primer sequences used for quantitative real-time PCR

Gene	Forward Primer	Reverse Primer
GAPDH	5‘CACCCACTCCTCCACCTTTG3'	5‘CCACCACCCTGTTGCTGTAG3'
Notch1	5’GTCAACGCCGTAGATGACC3’	5’TTGTTAGCCCCGTTCTTCAG3’′
Jagged1	5’GCTTGGATCTGTTGCTTGGTGAC3’	5’ACTTTCCAAGTCTCTGTTGTCCTG3’
Jagged2	5’ GCTATTTCGAGCTGCAGCTGAG3’	5’GCGGCAGGTAGAAGGAGTTG 3’
Delta 1	5’CTACACGGGCAGGAACTGCAG3’	5’CGCCTTCTTGTTGGTGTTCTTG3’

### Notch1 shRNA lentivirus transfection

Notch1 shRNA (shRNA-Notch1) and negative control (shRNA-NC) in pGLVl/U6/GFP lentiviral vectors (purchased from GenePharma Co,Ltd.) were prepared. The target sequence for Notch1 shRNA was 5’-GGAGCATGTGTAACATCAACA-3’. The shRNA-Notch1 lentiviral vectors were constructed according to manufactors suggestion. The Lin-CD45RA-cells were seeded at 5 × 104 cells/well into 96-well plates at 24-h prior to transfection. When the cells grew to 60–70 % confluenced, transfection was carried out by using lentiviral particles (Lin-CD45RA-DC precursors multiplicity of infection (MOI) = 200), polybrene (5 μg/ml), and enhanced infection solution (GeneChem Co., Ltd., Shanghai, China), according to the manufacturer’s protocol. At 12h post-transfection, virus-containing medium was replaced with complete medium. At 24h, 72 and 96h post-transfection, all cells were observed under fluorescence inverted microscope. The NOTCH 1 mRNA expression in different time was detected by RT-PCR.

### Treatment by γ-secretase inhibitor(GSI)

DAPT (N-[(3, 5-difluorophenyl) acetyl]-L-alanyl-2-phenyl] glycine-1,1-dimethylethyl) is a kind of GSI. It was bought from Sigma (USA). In the culture of Lin-CD45RA-DC precursors, DAPT was added in the culture system to inhibit the Notch pathway with initial cell density of 5 × 10^5^/ml, at concentrations of 2.5 to 20μM. Dimethyl sulfoxide (DMSO) (1:1000 dilutions) was added to the cell culture system as control. Notch1 mRNA expression was detected by qRT-PCR after cells were cultured with DAPT or DMSO for 72 h.

### Induced maturation of Lin-CD45RA-DC precursors in different culture condition

After being treated in different conditions, Lin-CD45RA- DC precursors, shRNA-Notch1 transfected cells and cells treated by DAPT were all seeded in 24-well plate separately by a density of 1-5 × 105/ml in 500 μl RPMI 1640 plus GM-CSF (50 ng/ml) and TNF-α (20 ng/ml) for another 5 days culture.

### Flow cytometry analysis

For three-color immunolabeling, 1 × 10^5^cells were labeled with mAb mixture of peridinin chlorophyll protein (PeCy5)-conjugated anti-HLA-DR, APC-conjugated anti-CD11c and phycoerythrin (PE)-conjugated anti-CD123. At least 100,000cells were analyzed on a BD FACS Arial II. All of the above mAbs were bought from BD Biosource, USA. Analysis of flow cytometry data was ebioscience performed using Expo32 software.

### Detection of IL-12 in DC culture supernatants

Human IL-12 was measured quantitatively in Cell-culture supernatants using the Human Interleukin 12 ELISA Kit (BlueGene Biotech CO.LTD) according to the instructions in the kit.

### Statistical analysis

The statistical significance was determined using 2-tailed unpaired Student’s t-test analysis using Prism5 software (GraphPad). The data are presented as the mean ± SD with significant difference as *p* < 0.05. Asterisks were used to denote *P* values as: not significant (ns) = *P* > 0.05; *P ≤ 0.05; **P ≤ 0.01; ***P ≤ 0.001.

## Results

### Notch1 receptors and ligands were detected by RT-PCR in Lin-CD45RA- DC precursors

**Fig. 1:**
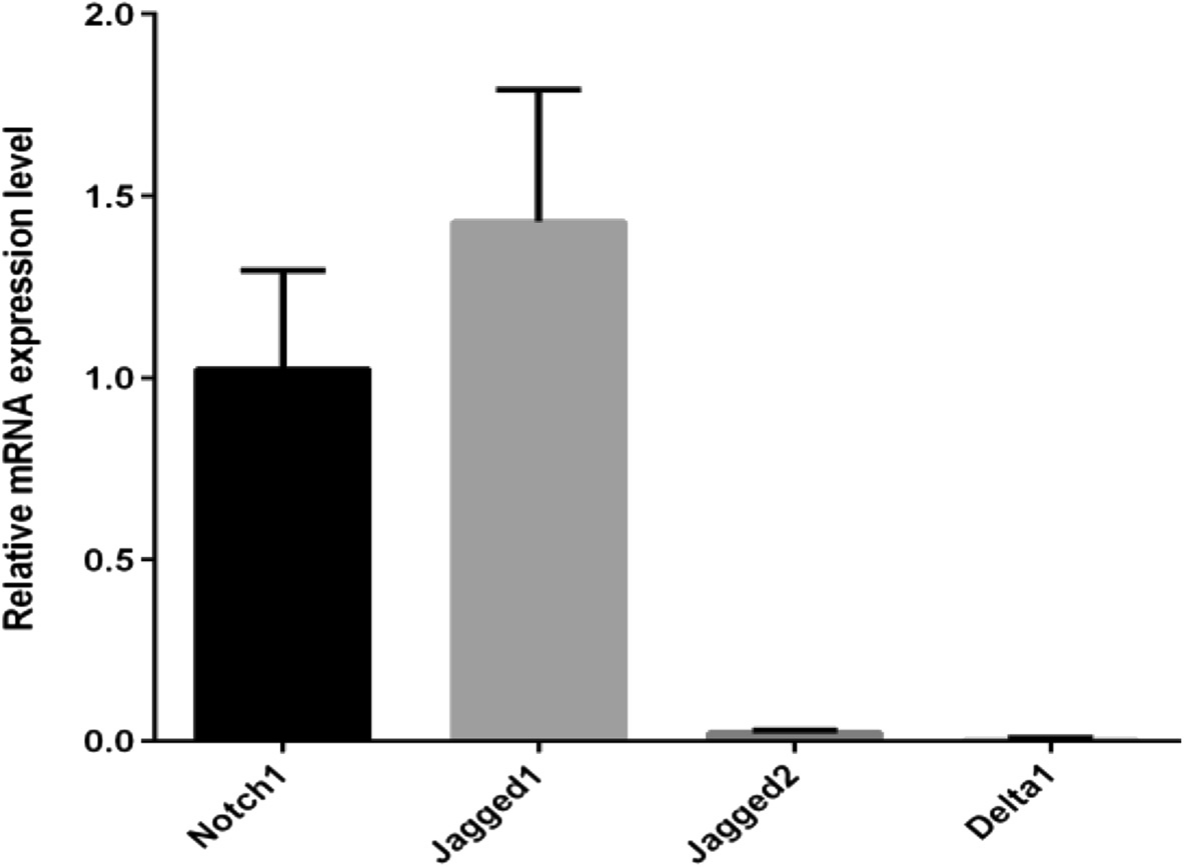
Real time PCR analysis of Notch 1, Jagged1, 2 and Delta1 in LIN-CD45RA- DC precursors (mean ± SD, *n* = 3)

In the Lin-CD45RA- DC precursors developed from CD34+ cells, we can detect the expression of Notch1 receptors and their ligands, Jagged 1, Jagged 2, and Delta 1 ligands. Among them,Notch1 receptor and Jagged1 ligand had high expressions, while expressions of Jagged2 and Delta1 were much lower (Fig. 1).

### Notch1 was knocked down by shRNA

**Fig. 2:**
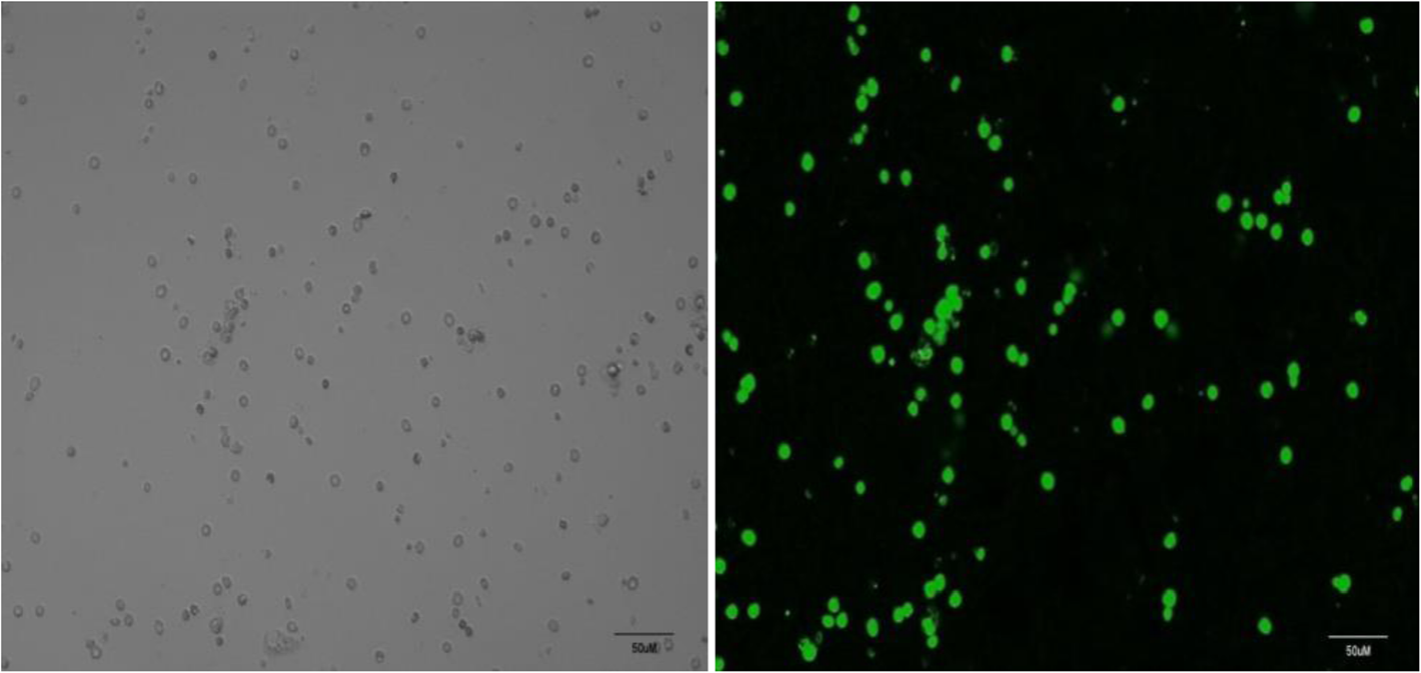
Both pictures showed Lin-CD45RA- cells, however the left picture 24h after transfection and the right picture 72h after transfection (1 × 10^9^TU/ml 1:4, ×200)

The shRNA-Notch1 lentiviral expression vector was constructed and we used it to transfect the Lin-CD45RA-DC precursors (MOI = 200). The LIN-CD45RA-DC cells had fluorescence after 24h lentivirus infection. The fluorescence demonstrated high transfection efficiency of over 90 % at 72h (1 × 10^9^TU/ml 1:4) (Fig. 2).

**Fig. 3:**
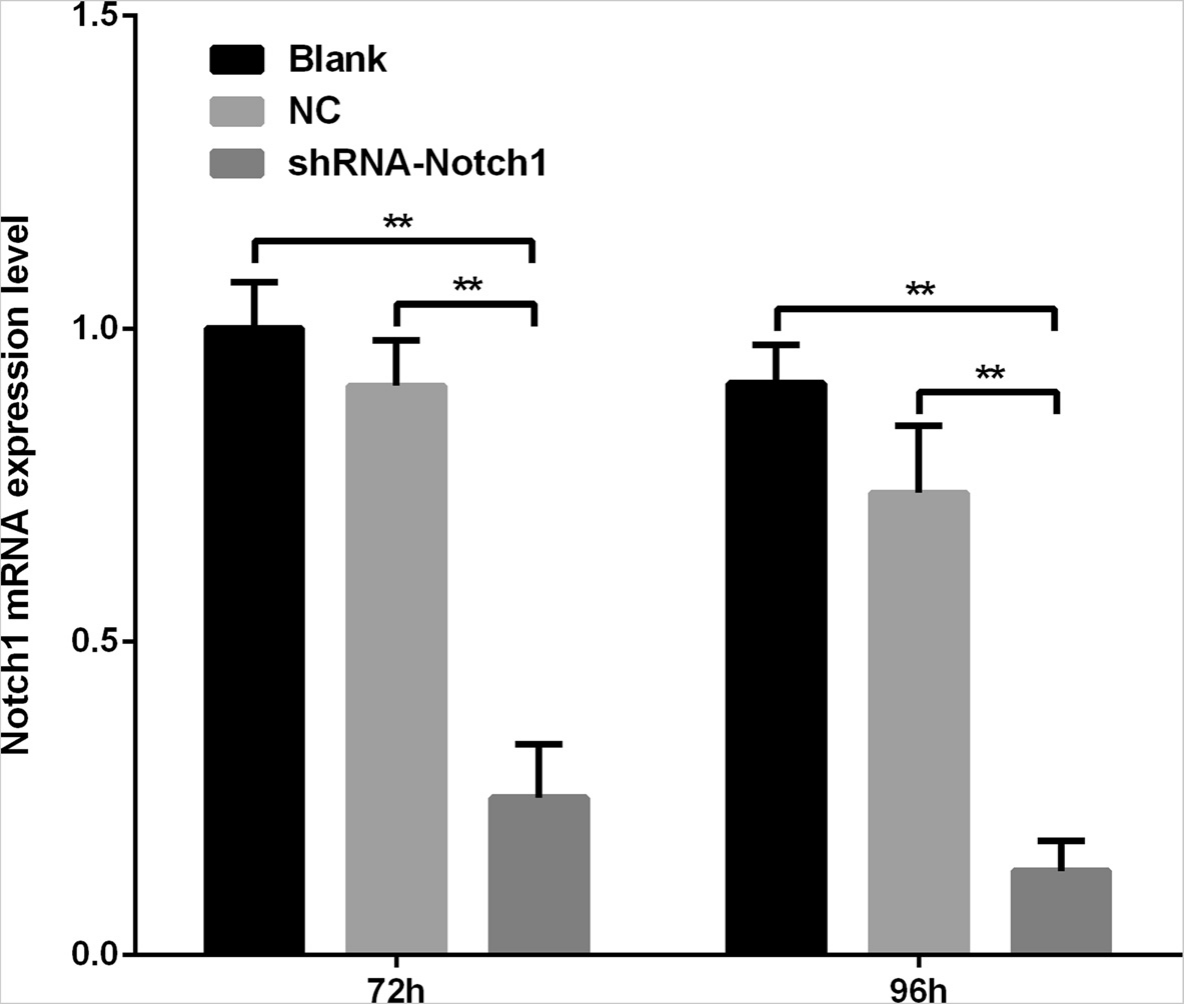
Real-time PCR showed that Notch1 mRNA level significantly decreased after 72h/96h of lentvirus infection in Lin-CD45RA-DC precursors. **P < 0.01

The relative expressions of Notch1 mRNA in the following groups of blank group, negative control group and shRNA-Notch1 group after 72h lentivirus infection were 1.002 ± 0.042, 0.909 ± 0.041 and 0.251 ± 0.049 respectively. The relative expressions of Notch1 mRNA after 96h lentivirus infection were 0.913 ± 0.035, 0.737 ± 0.062 and 0.133 ± 0.027 respectively. As compared to the blank group and negative control group, the ShRNA-Notch1 lentiviral expression vector significantly down regulated the expression of Notch1 mRNA in Lin-CD45RA-DC cells. However, there was no significant difference between the shRNA-Notch 1 lentiviral groups after 72h lentivirus infection and after 96h lentivirus infection (0.251 ± 0.049 vs 0.133 ± 0.027, *p* = 0.10) (Fig.3). In the following experiment, we used the cells after 72h lentivirus infection.

### Notch1 was knocked down by GSI

**Fig. 4:**
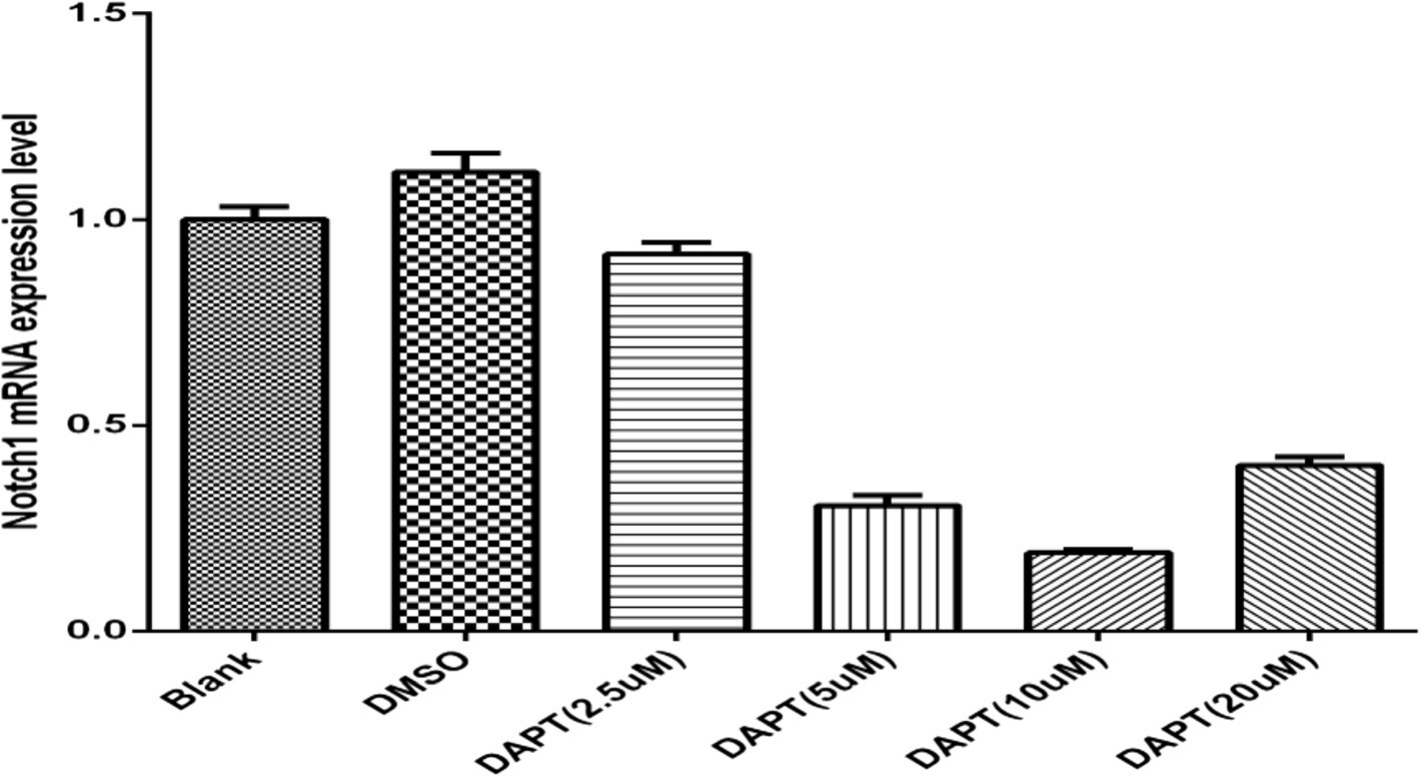
The mRNA relative expression of Notch1 after treated with DAPT for 72h (D1: DAPT 2.5μM; M: DAPT 5μM; D3: DAPT 10μM; D4: DAPT 20μM). With the increased concentration of DAPT, the expression of Notch1 decreased till the concentration increased to 20um. The concentration of 10μM showed the greatest inhibitory effect

Moreover, we also used DAPT to block the Notch1 signaling. According to the experiment, the expression of Notch1 mRNA was significantly reduced after treated with a certain concentration (5μM, 10μM, 20uM) at 72h. As a result, cells treated with 10μM DAPT had significant inhibition on Notch1 expression (Fig. 4).

**Fig. 5:**
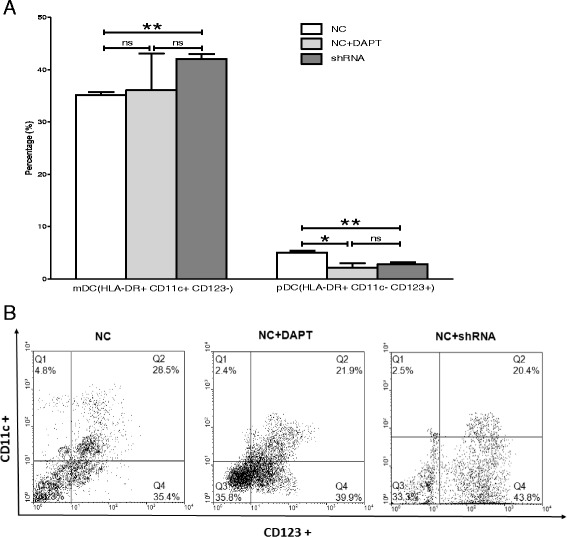
**a** Flow cytometry analysis showed the differentiation of Lin-CD45RA-DC precursors without SKOV3 culture supernatants (*n* = 3, mean ± SD, %). shRNA mediated Notch 1 knock down significantly differentiated more mDCs(*p* = 0.001), but generated less pDCs (*p* = 0.007). GSI mediated Notch 1 knock down significantly differentiated less pDCs (*p* = 0.015), but had no effect on mDCs (*p* = 0.45). **b** Representative flow histograms of DCs subtypes obtained in different culture conditions using a mAb mixture containing HLA-DR, CD11c, and CD123 mAb. The cells were identified on the basis of their surface expression of HLA-DR, and then DCs were further analyzed by CD11C and CD123 expression within the HLA-DR+ gate. mDCs and pDCs were defined as HLA-DR + CD11c + CD123- and HLA-DR + CD11c-CD123 +

### Notch 1 knockdown influenced the differentiation of Lin-CD45RA-DC precursors without SKOV3 cultured supernatants

We used 3-colour flow cytometry to analyze the differentiation of Lin-CD45RA- DC precursors in different conditions. As a result, after lentivirus transfection of shRNA-Notch1, when compared with control group, Lin-CD45RA-DC precursors differentiated into more HLA-DR + CD11c + CD123- mDCs(42.03 ± 0.98 % vs 35.17 ± 0.56 %, *p* = 0.001) and less HLA-DR + CD11c-CD123+ pDCs ( 2.76 ± 0.42 % vs 5.03 ± 0.33 %, *p* = 0.007).

As we showed above, DAPT down regulated the expression of Notch1 mRNA level. But when compared with control group, the differentiation of Lin-CD45RA-DC precursors to mDCs in DAPT group was almost the same (36.07 ± 6.99 % vs 35.17 ± 0.56 %, *p* = 0.45). Instead, significant decrease of pDCs was detected. The proportion of pDCs after GSI treating was 2.1 ± 0.8 %, and in control group was 5.03 ± 0.33 % (*p* = 0.015). There were no significant differences in the differentiation to either mDC or pDC between the shRNA group and GSI group (*p* = 0.22; *p* = 0.26) (Fig. 5a, Fig. 5b).

### Notch 1 knockdown influenced the differentiation of Lin-CD45RA-DC precursors within SKOV3 cultured supernatants

We have shown previously that SKOV3 cultured supernatants decreased the number of mDCs and increased the number of pDCs during the differential process of Lin-CD45RA-DC precursors. In order to verify the relationship of Notch1 signaling and the differentiation of DC precursors in the ovarian cancer microenvironment, we added SKOV3 cultured supernatants into different DC precursor culture system.

**Fig. 6:**
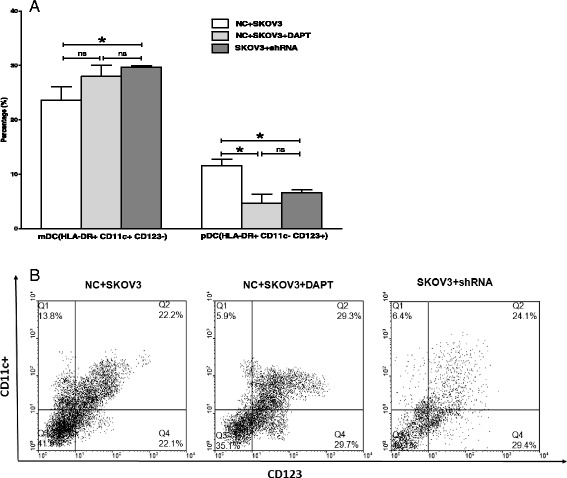
**a** Flow cytometry analysis showed the differentiation of Lin-CD45RA-DC precursors within SKOV3 culture supernatants(*n* = 3, mean ± SD, %). shRNA mediated Notch 1 knock down significantly differentiated more mDCs(*p* = 0.036), but generated less pDCs (*p* = 0.01). GSI mediated Notch 1 knock down significantly differentiated less pDCs (*p* = 0.013), but had no effect on mDCs (*p* = 0.45). **b** Representative flow histograms of DCs subtypes obtained in different culture conditions using a mAb mixture containing HLA-DR, CD11c, and CD123 mAb. The cells were identified on the basis of their surface expression of HLA-DR, and then DCs were further analyzed by CD11C and CD123 expression within the HLA-DR+ gate. mDCs and pDCs were defined as HLA-DR + CD11c + CD123- and HLA-DR + CD11c-CD123 +

When the DC-precursors were treated with SKOV3 supernatants, the differentiation to mDCs could also be significantly increased by shRNA-Notch1 (29.70 ± 0.21 % vs 23.57 ± 2.53 %, *p* = 0.036), but not by DAPT (27.97 ± 2.09 % vs 23.57 ± 2.53%, *p* = 0.125). However, the differentiation to pDCs could be significantly reduced by both shRNA and DAPT (6.57 ± 0.61% vs 11.57 ± 1.19 %, *p* = 0.01; 4.67 ± 1.65 % vs 11.57 ± 1.19 %, *p* = 0.013. respectively). There were no significant differences in the differentiation to either mDC or pDC between the shRNA group and GSI group (*p* = 0.22; *p* = 0.17) (Fig. 6a, Fig.6b).

### IL-12 concentrations in vitro

IL-12 is a DC-derived factor that directs the development of Th1 immune reaction. The capacity of DCs to produce bioactive IL-12p70 was detected and compared between groups treated with and without SKOV3-supernatants.

**Fig. 7:**
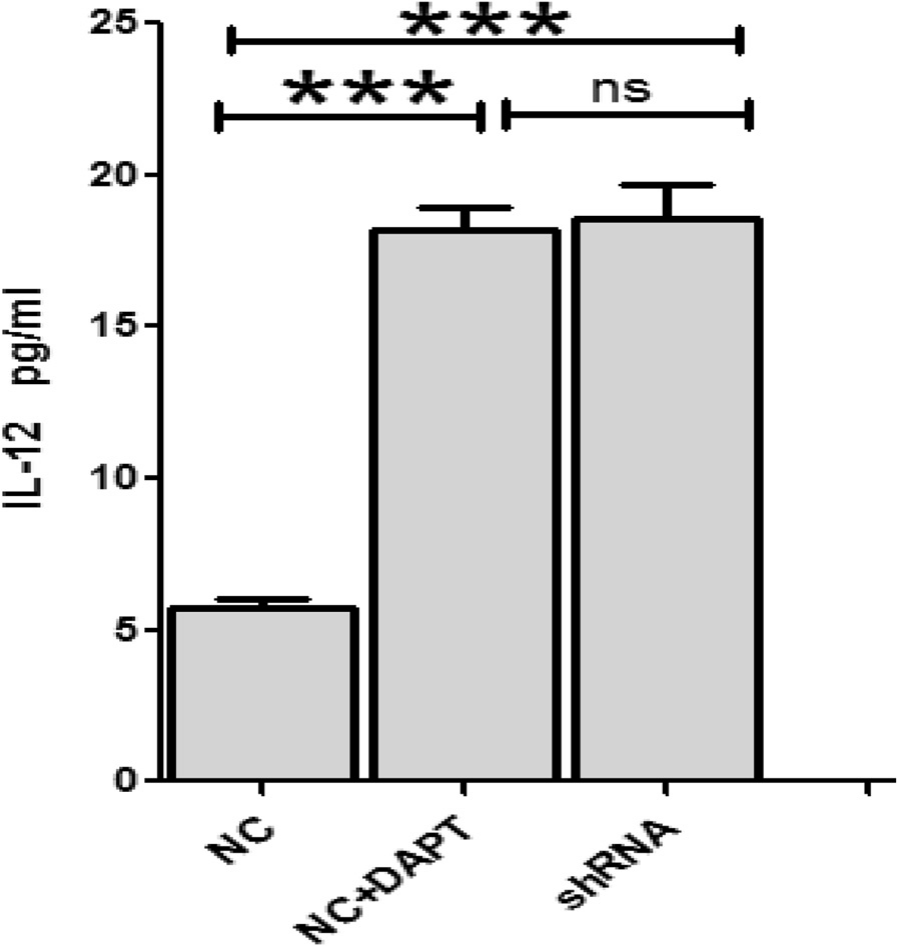
IL-12 concentrations in three culture supernatants without SKOV3 culture supernatants (*n* = 3, mean ± SD). Both shRNA and GSI mediated Notch 1 knock down significantly produced more IL-12 compared to control group (*p* = 0.0002, *p* = 0.0003, respectively)

In the groups without SKOV3-supernatants, after treated with either shRNA or DAPT, the secretion of IL-12 significantly increased as compared with the control group (18.54 ± 1.15 vs 5.72 ± 0.35, *p* = 0.0002; 18.16 ± 0.78 vs 5.72 ± 0.35, *p* = 0.0003 respectively) (Fig. 7).

**Fig. 8:**
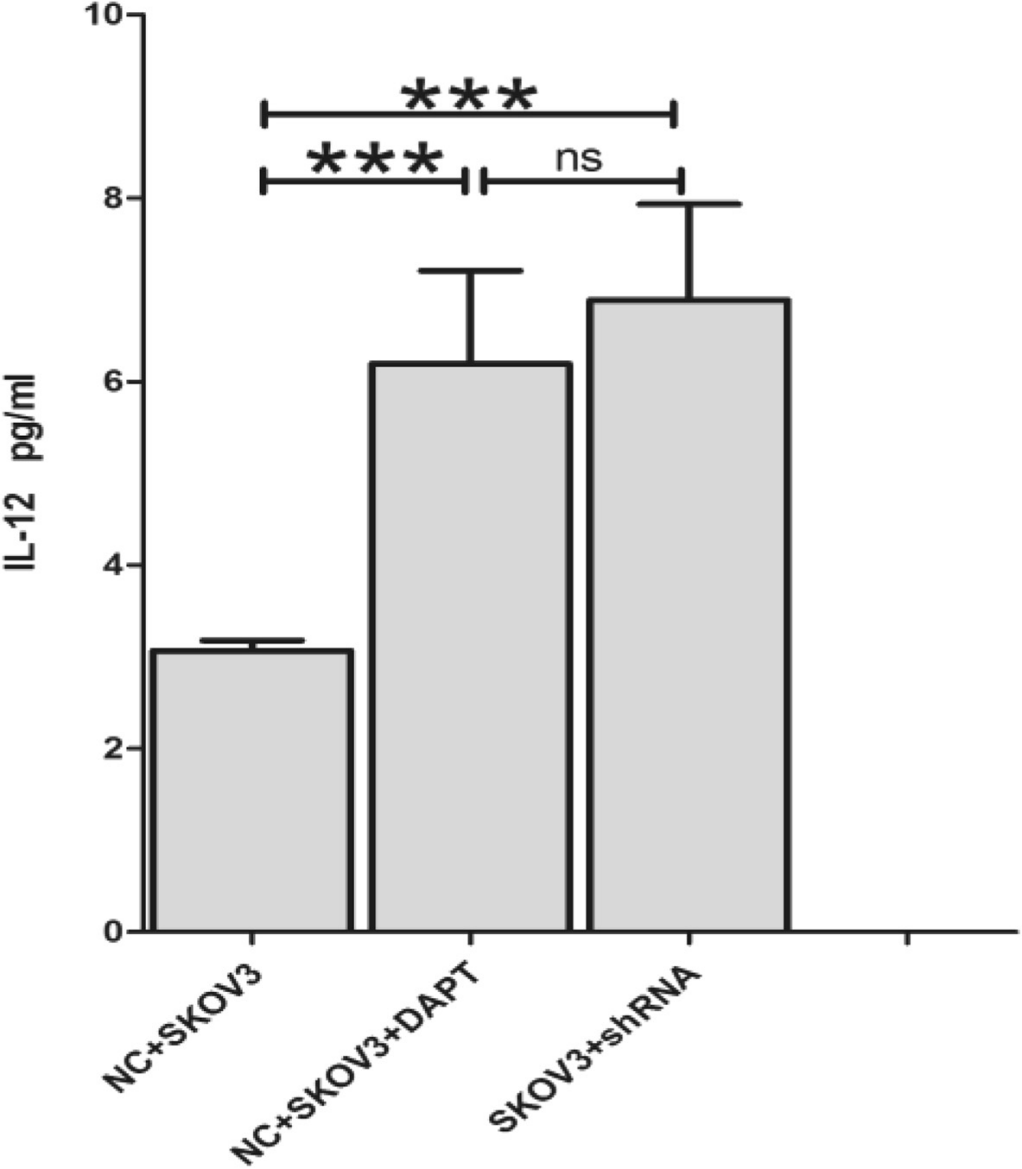
IL-12 concentrations in three culture supernatants within SKOV3 culture supernatants (*n* = 3, mean ± SD). Both shRNA and GSI mediated Notch 1 knock down significantly produced more IL-12 compared to control group (*p* = 0.011, *p* = 0.018, respectively)

The result is the same in the groups with SKOV3-supernatants, the secretion of IL-12 after treated with either shRNA or DAPT significantly increased as compared with the control group (6.89 ± 1.04 vs 3.06 ± 0.11, *p* = 0.011; 6.19 ± 1.01 vs 3.06 ± 0.11, *p* = 0.018. respectively) (Fig. 8).

## Discussion

There were three main findings of this study. Firstly, Notch1 and all the Notch family ligands were expressed in Lin-CD45RA-DC precursors. Secondly, the differentiation of Lin-CD45RA-DC precursor cells to pDCs can be reduced after knocking down Notch1 by both shRNA and DAPT, and this process would not be affected by the supernatant of the ovarian carcinoma cell line. Thirdly, the secretion of IL-12 significantly increased after knocking down Notch1 with or without the supernatants of SKOV3 cell line.

The previous studies, which explored the close relationship between Notch family and DCs differentiation, were controversial. It mainly concentrated on the following aspects. On the one hand, it is still not clear whether Notch1 would be a promotion or inhibition role in the differentiation of the DCs. Hoshino N et al. reported that Notch signaling was found to promote peripheral blood monocyte precursors differentiate to DCs and Langerhans cells [17]. However, De Smedt et al. found that inhibition of Notch signaling with γ-secretase inhibitor shifted differentiation into non-T cells including monocyte or dendritic cells [18]. Similar contradictory data exists in respect to the effect of Notch signaling on pDCs. Oliver et al. reported that Notch signaling via Delta-1 promoted differentiation of pDC [19]. In a different study, stromal cells expressing Delta-1 blocked pDC development [20]. On the other hand, which differentiation stage the Notch1 signal pathway would take effect is still unknown. Most studies were mainly focused on CD34+ hematopoietic progenitors and DCs stage. For instance,Els Waegemans et al. reported that blocking of Notch1 signaling at the stage of CD34+ hematopoietic progenitors resulted in a complete block in T-lineage specification and induced monocytic and plasmacytoid dendritic cells [21]. However, another study, which repealed that MicroRNA-23b promoted tolerogenic properties of dendritic cells in vitro through inhibiting Notch1/NF-jB signalling pathways, was mainly focus on the mature DC stage [22]. Additionally, the activation of Notch1 signaling by different ligands was still controversial. Cheng P et al. found that main Notch ligands Delta-1 and Jagged-1 had the opposite effect on DC differentiation. Delta-1 promoted generation of fully differentiated DCs, whereas Jagged-1 stimulated accumulation of DC precursors but prevented their transition to terminally differentiated DCs [23].

Notably, our present study was mainly discussing about the Notch1 expression in the Lin-CD45RA-DC precursors stage and its function in the transformation of pDCs. As we know, pDCs play an important part in the mechnism of immune escape, while mDCs secreted IL-12 would direct the development of Th1 cells and be in favor of anti-cancer immunity. In this study, we observed that the amount of pDCs was decreased but the level of IL-12 was increased in the supernatants of SKOV3 cell line after knocking down Notch 1. Therefore, it would be reasonable to hypothesize that Notch1 signaling inhibition may favor anti-tumor immune and it might provide a new target for immunotherapy of ovarian epithelial carcinoma.

Another most important and bewildering problem is that we observed a significant increase in myeloid differentiation after knock-down of the Notch1 by shRNA, while no such effect was found in GSI mediated Notch 1 knock-down. As a member of hydrolytic enzyme families, DAPT can hydrolyze Notch binding protein to block the Notch signal, and is also considered to have the effect to down regulate the Notch1 expression at the mRNA level [24]. We speculate that there might be other influential factors in the differentiation of mDCs. Therefore, further studies are still needed to confirm the differentiation mechanism of mDCs.

There are also some limitations in our study. For one thing, it may be appropriate to check for Delta1 and Jagged1 at the protein level for their activation to confirm the Notch 1 pathway in the differentiation of Lin-CD45RA-dendritic cell precursors. For another, it would be much better to check the knockdown effects of the canonical Notch target genes: Myc, p21, and the HES-family members. However, since CD34+ hematopoietic stem cells extracted from umbilical cord blood were relatively few, resulting in fewer Lin-CD45RA-DC precursors, then it is really becoming difficulty to complete the experiment simultaneously. Yet, it would be performed in our future experiments.

## Conclusion

In conclusion, we identified that Notch1 and Notch1 ligands were expressed in Lin-CD45RA-DC precursors. Notch1 is an important signaling pathway in the differentiation of Lin-CD45RA-DC precursor cells to plasmcytoid dendritic cells (pDCs). And this would not be affected by the supernatant of the ovarian carcinoma cell line. These findings might provide a new potential therapeutic target to ovarian cancer.

## Abbreviations

DAPT: N-[(3, 5-difluorophenyl) acetyl]-L-alanyl-2-phenyl] glycine-1,1-dimethylethyl
DC: dendritic cell
GSI: γ-secretase inhibitor
mDC: myeloid dendritic cell
pDC: plasmacytoid dendritic cell
